# Glycans in Sera of Amyotrophic Lateral Sclerosis Patients and Their Role in Killing Neuronal Cells

**DOI:** 10.1371/journal.pone.0035772

**Published:** 2012-05-30

**Authors:** Meital Edri-Brami, Benyamin Rosental, Dana Hayoun, Michael Welt, Hila Rosen, Itzhak Wirguin, Beatrice Nefussy, Vivian E. Drory, Angel Porgador, Rachel G. Lichtenstein

**Affiliations:** 1 Avram and Stella Goren-Goldstein Department of Biotechnology Engineering, Faculty of Engineering, Ben-Gurion University of the Negev, Beer-Sheva, Israel; 2 Shraga Segal Department of Microbiology and Immunology, Faculty of Health Sciences, The Cancer Research Center and The National Institute of Biotechnology, Ben-Gurion University of the Negev, Beer-Sheva, Israel; 3 Department of Neurology, Soroka Medical Center, Ben Gurion University, Beer Sheva, Israel; 4 Department of Neurology, Tel Aviv Sourasky Medical Center and Sackler School of Medicine, Tel Aviv University, Tel Aviv, Israel; Johns Hopkins University, United States of America

## Abstract

Amyotrophic lateral sclerosis (ALS) is a fatal neurodegenerative disease caused by degeneration of upper and lower motor neurons. To date, glycosylation patterns of glycoproteins in fluids of ALS patients have not been described. Moreover, the aberrant glycosylation related to the pathogenesis of other neurodegenerative diseases encouraged us to explore the glycome of ALS patient sera. We found high levels of sialylated glycans and low levels of core fucosylated glycans in serum-derived N-glycans of patients with ALS, compared to healthy volunteer sera. Based on these results, we analyzed the IgG Fc N^297^-glycans, as IgG are major serum glycoproteins affected by sialylation or core fucosylation and are found in the motor cortex of ALS patients. The analyses revealed a distinct glycan, A2BG2, in IgG derived from ALS patient sera (ALS-IgG). This glycan increases the affinity of IgG to CD16 on effector cells, consequently enhancing Antibody-Dependent Cellular Cytotoxicity (ADCC). Therefore, we explore whether the Fc-N^297^-glycans of IgG may be involved in ALS disease. Immunostaining of brain and spinal cord tissues revealed over-expression of CD16 and co-localization of intact ALS-IgG with CD16 and in brain with activated microglia of G93A-SOD1 mice. Intact ALS-IgG enhanced effector cell activation and ADCC reaction in comparison to sugar-depleted or control IgG. ALS-IgG were localized in the synapse between brain microglia and neurons of G93A-SOD1 mice, manifesting a promising *in vivo* ADCC reaction. Therefore, glycans of ALS-IgG may serve as a biomarker for the disease and may be involved in neuronal damage.

## Introduction

Immunoglobulins, the major secretory products of the adaptive immune system, include the glycoprotein IgG subclass, which identifies and neutralizes foreign cells [1]. As adaptors, IgG activate an immune response by simultaneously binding antigens through their variable domains (F(ab)2) and through interaction of their Fc domain with Fcγ receptors (FcγR) on immune cells. The human FcγR family consists of the activating receptor FcγRIIIA (CD16) that mediates antibody-dependent cellular cytotoxicity (ADCC) [2]. The binding capacity of IgG to CD16 was found to be lost after cleaving or preventing glycosylation at a single site on asparagine 297 (N^297^) in the IgG Fc domain [3]. The nature of the glycans attached to N^297^ affects the affinity of the CD16 interaction and thus governs antibody cytotoxicity [4]. It has been suggested that IgG play a role in motor neuron degeneration [5], [6]. This was based on the finding of IgG deposits on the spinal cord and brain of patients with amyotrophic lateral sclerosis (ALS) and in animal models of inherited ALS. It was further found in animal models that IgG from ALS patients could not be uptaken by motor axon terminals after removal of the IgG Fc domain [7]. Consequently, it appears that FcγRs are involved in IgG deposition or in uptake by motor neurons.

ALS is a fatal neurodegenerative disease caused by degeneration of the upper and lower motor neurons [8]. ALS patients and animal models of inherited ALS, like mutant Cu/Zn superoxide dismutase (mSOD1), display similar inflammatory responses at the site of the motor neuron injury, enabling both the CNS resident and systemic inflammatory cells to balance between neuroprotection and neurotoxicity [8]–[13]. Among others, microglia cells are activated during these inflammatory responses, changing their cell morphology and surface receptor expression, and producing growth factors and cytokines, leading to neuron protection or injury depending on the physiological conditions [14]. The manners in which the signals switch between protective to cytotoxic microglia are not yet fully understood. However, ALS progression is attributed, in part, to cytotoxic microglia cells, which secrete proinflammatory cytokines leading to neuron damage. Cumulative data demonstrate that Toll-like receptors or T-cells interacting with microglia are involved in inducing cytotoxic microglia [15], but no direct evidence has been found hitherto linking FcγR to microglia activity in ALS. Notably, in other neurodegenerative diseases such as Alzheimer's, there is evidence that the FcγRs are linked to phagocytosis by activated microglia [16].

**Table 1:** Statistic analyses comparing N-glycans derived from 19 ALS patient sera and from 24 healthy candidate sera.

Peak no.	Assignment	ANOVA (p Value)	Mann-Whitney (p Value)	Welch T-test (p Value)	Fold Change
1	A2	0.578	0.230	0.605	−1.13
2	Fc(6)A2	0.277	0.123	0.238	−1.09
3	Fc(6)A1G1	0.434	0.317	0.432	−1.09
4	Fc(6)A2G1	0.058	0.047	0.043	−1.15
	Fc(6)A2BG1				
	Fc(6)M4A1G1				
5	A2G2	0.118	0.063	0.140	+1.83
	M6A1				
6	Fc(6)A2G2	0.008	0.020	0.003	−1.32
	Fc(6)A2BG2				
	Fc(6)M6A1				
7	M7	0.480	0.511	0.550	+1.12
	A2G2S1				
	Fc(6)A2G2S1				
	Fc(6)A2BG2S1				
8	Fc(6)A2BG1S1	0.012	0.014	0.009	−1.16
	A2G2S1				
	Fc(6)A2G2S1				
9	M8	0.094	0.086	0.064	+1.12
	Fc(6)A2G2S1				
	Fc(6)A2BG2S1				
	A2G2S2				
10	A2G2S2	0.023	0.007	0.019	+1.36
	Fc(6)A2G2S2				
	Fc(6)A2BG2S2				
	A2F1G2S1				
	A3G3S1				
11	M9	0.039	0.079	0.024	−1.53
	A3G3S1				
	A3BG3S1				
	A4G3S1				
12	A3G3S2	0.257	0.193	0.262	−1.22
	Fc(6)A3G3S1				
	A4G3S1				
	A3F1G3S1				
13	A3G3S3	0.001	0.001	0.001	+1.67
	Fc(6)A3G3S2				
	A4G3S2				
	A3F1G3S2				
	Fc(6)A3F1G3S1				

Fold of change: (+) assigns that N-glycan amounts in ALS sera are higher relative to glycans from healthy candidate sera and (−) assigns the contrary.

We propose that over-expression of CD16 on activated microglia or infiltrating immune cells can increase the incidence of binding ALS-produced IgG through an Fc glycan, A2BG2, thus inducing neuron loss. Here we tested this hypothesis by first applying the N-glycome approach and then by demonstrating over-expression of CD16 and co-localization of ALS-IgG with CD16 in sections of brain and spinal cord tissues from 130- and 75-day old G93A-SOD1 mice. Additionally, we demonstrated *in vitro* activities of intact ALS-IgG with this Fc glycan, including its role in ADCC against neuroblastoma and neuroblastoma-spinal cord motoneuron hybrid cell lines. Finally, we showed localization of intact ALS-IgG in the immunological synapse between microglia and the neuron of G93A-SOD1 brain tissue, thus suggesting the occurrence of *in vivo* ADCC.

## Materials and Methods

### Ethics Statement

The study was performed in conformity with the Declaration of Helsinki, and approved by the Ethics Committee of Ben-Gurion University and of Tel-Aviv University. All subjects gave written informed consent prior to participation.

**Figure 1:**
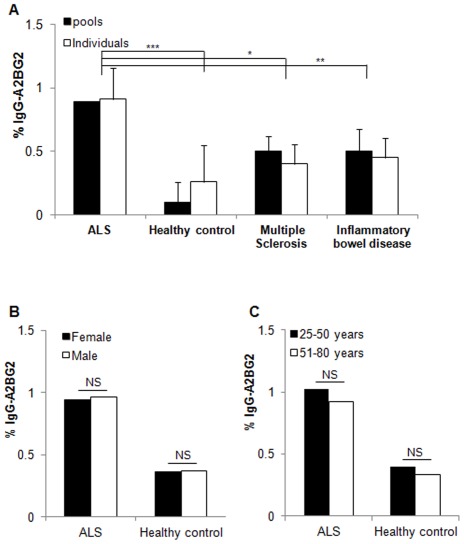
The Fc-N^297^-glycan of ALS-IgG contains a galactosylated glycan with bisecting-GlcNAc and lacking a core fucose. ANOVA t-test analysis of glycan amounts in individual or in pooled serum samples of healthy controls and inflammatory bowel disease, multiple sclerosis and ALS patients The glycan amounts and structures were determined by NP-HPLC and MALDI-TOF MS methods (**A**), ANOVA t-test analysis of A2BG2 with respect to gender (**B**) and age (**C**). Data represent means ± SD of measurements of 19 ALS, 24 healthy controls, 22 inflammatory bowel disease patients, and 6 multiple sclerosis patients or 6 pools generated by mixing four individual serum samples from every group (in multiple sclerosis two pools were generated by mixing three individual serum samples). Statistical significance, *** p<0.005, ** p<0.01 and *, p<0.05, versus the appropriate control.

### Human subjects

Sera were collected from 19 ALS patients (11 males). The average patient age at sampling was 60±13 years (range 28–80) and the average duration of the disease was 26±13 months (range 9–54). Two patients had familial ALS. There was a relatively high incidence of bulbar-onset patients in our sample (9–47%). The average disability of the patients at the time of the examination, as measured by the ALSFRS-R score was 34±7 (range 17–44).

**Table 2:** Statistic analyses comparing N^297^-glycans digested from serum IgG of 19 ALS patients and of 24 healthy candidates.

Peak no.	Assignment	ANOVA (p-Value)	Mann-Whithney (p-Value)	Welch T-test (p-Value)	Fold Change
1	A2	0.9	0.693	0.9	−1.03
	Fc(6)A1				
2	Fc(6)A2	0.906	0.717	0.906	−1.02
3	Fc(6)A2B	0.768	0.974	0.768	−1.05
4	A2G1	0.288	0.293	0.289	−1.19
5	Fc(6)A2G1	0.027	0.038	0.029	−1.1
	Fc(6)A2BG1				
	A2BG1				
6	A2G2	0.797	0.717	0.797	+1.04
7	A2BG2	0.0027	0.004	0.0033	+2.1
8	Fc(6)A2G2	0.276	0.341	0.281	−1.12
9	Fc(6)A2BG2	0.737	0.669	0.738	−1.06
10	A2G2S1	0.254	0.178	0.259	+1.18
11	A2G2S2	0.473	0.622	0.474	+1.15
12	Fc(6)A2G2S1	0.14	0.450	0.152	+1.6
13	Fc(6)A2G2S2	0.202	0.237	0.215	+2.9

Fold of change: (+) assigns that N^297^glycan amounts in ALS IgG are higher relative to N^297^glycans from healthy control IgG and (−) assigns the contrary.

For control experiments, sera were collected from 24 healthy subjects (13 males) with an average age of 48±11 years. As non-ALS disease controls, sera were taken from 22 patients (12 males) with inflammatory bowel disease, with an average age of 44±14 years. Ten patients had Crohn's disease and the rest suffered from ulcerative colitis. As an additional control for neurodegenerative disease, sera were taken from 6 patients with multiple sclerosis with an average age of 42±17 years.

### Blood collection and analysis

The collected blood samples were collected in 10 ml SST Vacutainers (BD Biosciences, San Jose, CA) and allowed to clot at 4°C for 30 min. Coagulated blood was centrifuged at 3,000 rpm for 10 minutes, and the serum portion was immediately divided into 50 µl aliquots in low-binding vials and frozen for storage at −80°C until thawed for glycomic and immunologic analyses. The aliquots were coded by the Sourasky hospital, where the sample information was not available to the laboratory research assistants. Two research assistants carried out the glycomic experiments. One assistant used the individual and pooled samples, the other used the individual samples, providing two independent experiments.

### Glycan separation and normal phase HPLC analysis

In accordance with previous procedure [17], PNGase-F-released glycans (Roche Diagnostics, Germany) from 50 µl of serum were fluorescently labeled with 2-aminobenzamide (2-AB), by reductive amination. The glycans were subsequently separated on a 4.6×250 mm Glyco-Sep N column (Waters, Milford, MA) using two Waters 510 pumps, a Waters 717 auto-injector, and a FP-920 fluorescent detector (Jasco, Easton, MD). The solvents used were buffer A (50 mM ammonium formate, pH 4.4) and buffer B (acetonitrile). The glycans were eluted by a linear gradient of buffer A, such that initial conditions were 20% buffer A at a flow rate of 0.4 ml/min. The concentration of buffer A was changed from 35–53% (the remainder was buffer B) over 132 min, and then from 53–100% over the next 3 min, at a constant flow rate. The column was washed with 100% buffer A for 5 min at a flow rate of 1 ml/min before re-equilibration in the initial solvent system. In order to determine the glycan structures, eluted glycans from individual samples were collected manually according to retention time, concentrated in a speed vacuum, and finally pooled into ALS and control samples. Likewise, eluted glycans from 19 ALS patients and 24 healthy control samples were numbered according to retention time and their amounts were calculated by Empower software (Waters). Glycans were assigned glucose unit (GU) values, and their structures were predicted by comparison to a glycan database made available for use in this analysis.

**Figure 2:**
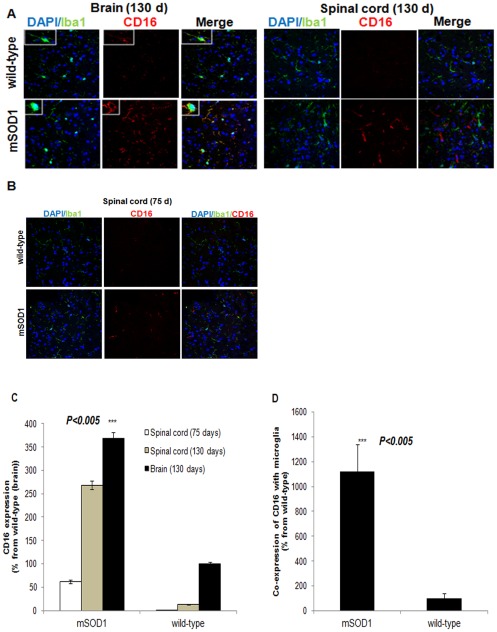
Expression and co-expression of CD16 with microglia of G93A-SOD1 brain and spinal cord tissues. Representative confocal microscopic images of brain cortex and spinal cord slices taken from 130-day old G93A-SOD1 mice and age-matched littermates stained for CD16, Iba1 (microglia), and counterstained with nuclear DAPI (**A**) The boxed area in A is a high magnification of CD16-positive microglia (small images in the merged images, **A, left**); Spinal cord slices taken from 75-day old G93A-SOD1 mice and age-matched littermates stained similarly as described in A (**B**); Expression of CD16 in brain and spinal cord tissues of mSOD1 in comparison to wild-type mice (in 8 µm brain slices) (**C**); Co-expression of CD16 with microglia in mSOD1 relative to wild-type mice (**D**). The quantity of CD16 was analyzed by measuring red intensity per defined area and the quantity of CD16 co-expressed with Iba1 was analyzed by measuring % of red intensity on a defined green intensity area. The measurements were performed on five fields from 3–4 sections per mouse. Error bars indicate means ± SD. The P value analysis *** p<0.005, ** p<0.01 and * p<0.05, versus non-SOD1 littermates represent a comparison with an ANOVA t-test.

**Figure 3:**
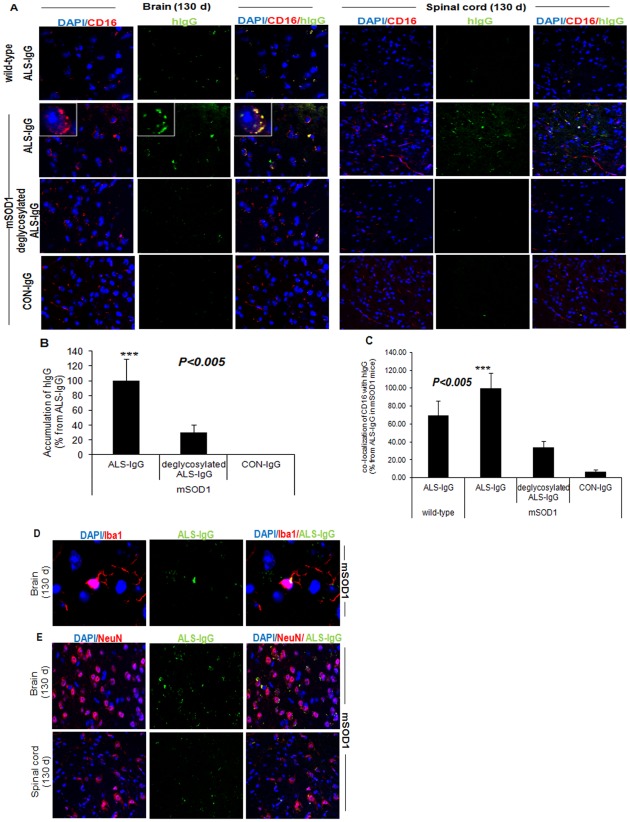
ALS-IgG co-localized with CD16 and microglia cells in brain and spinal-cord tissues of G93A-SOD1 mice. Representative confocal microscopic images of brain cortex and spinal cord slices taken from 130-day old G93A-SOD1 mice and age-matched littermates stained for CD16, hIgG, Iba1, NeuN (neurons) and counterstained with nuclear DAPI. Localization of ALS-IgG before and after PNGase-F treatment and of healthy control-IgG in wild-type and mSOD1 brain tissues. The boxed area is a high magnification of CD16- and intact ALS-IgG-positive cell (**A**); Quantity of ALS-IgG accumulation before and after PNGase-F treatment and of IgG from healthy control in mSOD1 brain slices (**B**); Quantity of ALS-IgG before and after PNGase-F treatment and of IgG from healthy control co-localized with CD16 in wild-type and mSOD1 brain tissues (**C**); Co-localization of intact ALS-IgG with microglia was detected in mSOD1 brain tissue (**D**), and co-localization of intact ALS-IgG with NeuN in mSOD1 brain and spinal cord tissues (**E**). The measurements were performed on five fields from 3–4 sections per mouse. Error bars indicate means ± SD. Asterisks denote the significance of differences relative to deglycosylated ALS-IgG or control-IgG in mSOD1 sections or ALS-IgG in non-SOD1 littermates, *** p<0.005 represents a comparison using ANOVA t-test.

### Exoglycosidase digestions

Exoglycosidase digestion was used to define the structures of glycans present in the pooled and the individual samples, in conjunction with HPLC. A series of exoglycosidases supplied by Prozyme (San Leandro, CA) was applied to the 2-AB-labeled N-glycans to remove their terminal sugar residues. The digestions were conducted using 50 mM sodium acetate buffer, pH 5.5, for 16 h at 37°C, at the following concentrations: 1 U/ml *Arthobacter ureafaciens* sialidase (ABS); 1 U/ml bovine testis β-galactosidase (BTG); 120 U/ml *Streptococcus pneumonia* β-hexoaminidase (SPH); 100 U/ml bovine kidney fucosidase (BKF); and 100 mU/ml jack bean α-mannosidase.

### IgG purification

Serum IgG from ALS, inflammatory bowel disease, and multiple sclerosis patients, as well as from healthy controls were purified using protein G sepharose beads according to the manufacturer's instructions (GE healthcare, Germany). Briefly, 1 volume of serum (50 µl) was diluted with 1 volume of binding buffer (20 mM sodium phosphate, pH 7.0) and applied onto a protein G column. After 1 h of incubation at room temperature under rotating conditions, the beads were washed, the IgG fraction was eluted with 100 µl of elution buffer (0.1 M glycine-HCl, pH 2.7), and the supernatants were collected into 1 M Tris-HCl, pH 8.5, to neutralize the IgG solutions to pH 7.5. IgG concentration was determined by Bradford assay (Bio-Rad, Hercules, CA). IgG molecules were purified from individual serum samples (total of 71 samples), or from pool samples; a typical pool sample consisted of a balanced-serum mixture of four individuals.

### Digestion of IgG Fc N-glycans

IgG molecules were reduced in Laemmli sample buffer (Bio-Rad) and 50 mM DTT for 10 min at 70°C, followed by loading on one dimensional SDS-PAGE gels (10%). The gels were run in a MiniProtean3 device (Bio-Rad) for 75 min at 140–170 mV, at a current lower than 350 mA [18]. The IgG molecules were visualized with Coomassie blue stain and the relevant bands were excised, cut into small pieces and dried using vacuum centrifugation. In accordance with previous procedure [17], three units of PNGase F diluted in 27 µl of 20 mM NaHCO_3_, were added per 15 mm^3^ of gel and incubated for 16 h at 37°C. N-glycans were extracted from the gel pieces by collecting the supernatants of sequential gel incubations with 3×200 µl double distilled water (DDW), 200 µl acetonitrile (ACN), 200 µl DDW and finally 200 µl ACN in a sonicating water bath for 30 min at room temperature. The collected supernatants were concentrated to a volume of 500 µl and then decontaminated using AG-50 (H^+^ activated) ion-exchange resin. The glycans were dried for fluorescent labeling and HPLC analysis.

### N-glycan analysis by matrix-assisted laser desorption ionization time-of-flight mass spectrometry (MALDI-TOF MS)

The HPLC-eluted glycans were collected according to retention time, concentrated in a speed vacuum and finally pooled into ALS and control samples. The samples were diluted in 1 µl DDW, desalted on a nafion membrane at room temperature for 30 min and mixed with equal volumes of saturated 2,5-dihydroxy benzoic acid (DHB) solution (Bruker Daltonics, Bremen, Gemany), 50% ACN and 50% trifluoroacetic acid (TFA). The mixtures were analyzed by MALDI-TOF MS (Bruker Daltonics). A peptide calibration standard (Bruker Daltonics) was used to calibrate all spectra.

### Removing N^297^ glycans from IgG

IgG N^297^ glycans were removed by loading 50 µl of serum pooled from ALS patients onto protein G sepharose beads for 1 h at room temperature, with slow rotation. The beads were washed with glycan digestion buffer containing 0.01 M NH_4_HCO_3_, pH 8.5, followed by incubation with 0.5 U PNGase-F for 16 h at 37°C, under rotation conditions. The supernatant containing the digested glycans was removed and the N-glycan-free IgG molecules (deglycosylated IgG or PNGase F-treated IgG) were collected after elution and neutralization, as described above. The digestion was confirmed by one dimensional SDS-PAGE gels (10%) and immunoblot using *Erithrina cristagalli* lectin (ECL, Vector, Burlingame, CA), as previously described [18].

### Cell cultures

The human SHSy5y neuroblastoma (CRL2266, ATCC, Manassas, VA), HeLa (CL-2, ATCC) and PANC1 (CRL1469, ATCC) cell lines and the mouse neuroblastoma-spinal cord motoneuron hybrid cell line (NSC 34, a generous gift from Prof. D. Offen,) were grown in Dulbecco's modified Eagle's medium (DMEM) supplemented with 10% (v/v) fetal calf serum (FCS), 100 units/ml penicillin, 0.1 mg/ml streptomycin, and 2 mM L-glutamine, in a humidified 5% CO_2_ atmosphere at 37°C.

The murine BW thymoma cell line, CD16-stable transfectants of BW cells [19], and the THP1 cell line (TIB-202, ATCC) were cultured in complete Roswell Park Memorial Institute medium (RPMI-1640) supplemented with 10% (v/v) heat inactivated FCS, 100 units/ml penicillin, 0.1 mg/ml streptomycin, and 2 mM L-glutamine in a humidified 5% CO_2_ atmosphere at 37°C, all from Invitrogen (Carlsbad, CA).

### Isolation of PBMCs and enriched peripheral NK cells

Human primary peripheral blood mononuclear cells (PBMCs) were purified from whole blood of healthy donors using Ficoll-Paque PLUS (GE Healthcare) according to the manufacturer's instructions. For isolation of a NK cell subset, PBMCs were purified from buffy coat extractions from healthy donors. NK cell subsets were purified by negative selection using antibody-coated magnetic bead separation (Invitrogen), following the manufacturer's instructions. Cell subset purity was assessed by flow cytometry, and determined to be 90%. The PBMCs or NK cells used as effector cells were activated by culturing in 10% heat inactivated FCS in RPMI-1640 containing 10 U/ml IL-2 (eBiosciences, San Diego, CA) overnight.

### Flow cytometry

To determine IgG affinity to SHSy5y cells, 1×10^5^ cells were harvested and blocked with FACS buffer (2% FCS and 0.05% sodium azide in PBS, pH 7.5) (Biolegend, San Diego, CA). Subsequently, the cells were incubated with pools of serum samples or protein G-enriched IgG of ALS, inflammatory bowel disease, multiple sclerosis patients or healthy controls at a dilution of 1∶10. Goat anti-human IgG allophycocyanin-conjugated F(ab′)_2_ (Jackson Immuno-Research, West Grove, PA) was used as a second step reagent. Propidium iodide (PI) was used to stain dead cells. For blocking CD16 on BPMCs, 1×10^5^ cells were incubated in FACS buffer and purified anti-human CD16 antibodies (Biolegend). Subsequently, the cells were washed and incubated with pools of serum samples of ALS patients. Goat anti-human IgG conjugated with allophycocyanin (Jackson Immuno-Research) was used as a second step reagent. Flow cytometry was performed using a FACS Calibur flow cytometer (BD Biosciences), and fluorescence data were acquired using logarithmic amplification. Data files were acquired and analyzed using BD CellQuest 3.3 software.

### Measurement of secreted cytokines

U-shape 96 well plates were incubated with serum, protein-G-enriched IgG or serum-depleted IgG samples for 3 h in a humidified 5% CO_2_ atmosphere at 37°C. After IgG absorption, the plates were intensively washed and incubated with 1×10^5^ healthy donor-enriched NK cells or CD16-transfected and control BW cells for 18 h in a humidified 5% CO_2_ atmosphere at 37°C. The media were collected and levels of secreted human IFNγ and mouse interleukin IL-2 were assessed using a commercial ELISA kit (Biolegend), according to the manufacturer's instructions and as described [19].

### ADCC assay

Antibody-dependent cytolytic activity was evaluated in co-cultures of neuroblastoma and NSC34 cells with serum or purified IgG from the different samples and PBMCs in a 4 h CFSE/7-AAD flow cytometry assay, as previously described [20]. Neuroblastoma and NSC34 cells, serving as target cells, were pre-incubated with diluted sera (1∶10) or with purified ALS-IgG before and after removing N^297^ glycans, for 1 h on ice. The cells were washed in RPMI medium and plated at a density of 5×10^4^ cells per well of a 96-well plate. The isolated PBMCs were pre-labeled with CFSE (Invitrogen) and co-cultured with the complex IgG-target cells at effector/target (E/T) cell ratios of 10∶1, in a final volume of 200 µl RPMI medium at 37°C for 4 h in a humidified CO_2_ incubator. To measure spontaneous lysis, target cell cultures were used. For cytolytic activity independent of IgG, target cells were co-cultured with PBMCs. For measuring lysis of target cells by IgG, target cells were incubated with IgG or serum samples. To confirm that the ADCC response occurred via NK cells, the PBMCs were pre-treated with anti-CD16 antibodies (BD Biosciences) for 30 min on ice and then co-cultured with target cells before coupling with the IgG. Killing assays were performed in quadruplicates. Acquisition was performed immediately afterwards on a FACScan flow cytometer equipped with a single 488 nm Argon laser. CFSE fluorescence and 7-AAD emission were detected in the FL-1 and FL-3 channels, respectively. Analysis was performed with FlowJo software (Three Star, Ashland, OR).

### G93A-SOD1 mice and frozen sections

Frozen sections of brain and spinal cord tissues of G93A-SOD1 transgenic and wild-type mice were kindly donated by Prof. D. Offen from Rabin Medical Center-Beilinson Campus, The Sackler School of Medicine at Tel-Aviv University and from Prof. M. Schwartz from The Department of Neurobiology at The Weizmann Institute of Science. The G93A-SOD1 transgenic mice were purchased from the Jackson Laboratory and were bred and maintained in the animal breeding center of the Tel-Aviv University (Approval ID 034-b328-4) and of the Weizmann Institute (IACUC; permission number 03680806-1). All experiments and procedures were approved by the Animal Care and Use Committee of Tel-Aviv University and of the Weizmann Institute.

For this experiment, G93A-SOD1 transgenic mice, 130 day old (the end-stage of the disease) and 75 day old [21] and wild-type age-matched littermates were deeply anesthetized, decapitated, and perfused with isotonic saline followed by 4% paraformaldehyde via cardiac puncture. Brain and spinal cord organs were immediately equilibrated in a 30% sucrose solution for 24 h, embedded in a frozen tissue matrix (Tissue-Tek OCT, Torrance, CA), cryo sectioned and stored at −80°C until section staining.

### Immunohistochemistry

Tick slides of 8 µm were blocked in PBS containing 1% BSA for 1 h and stained with mouse anti-neuronal nuclear protein (NeuN; 1∶25, GeneTex, Irvine, CA), mouse anti-CD16 (ASH 1975; 1∶50, Santa-Cruz Biotechnologies, Santa Cruz, CA), rabbit anti- ionized calcium binding adaptor molecule 1 (Iba-1; 1∶1000, Chemicon, Billerica, MA) antibodies, protein-G-purified ALS-IgG treated or untreated with PNGase F, and healthy volunteer IgG, at concentrations of 16 µg/ml. Anti-mouse CY5-, anti-rabbit CY3-, anti-human FITC-tagged or anti-mouse dyelight 549 secondary antibodies with low cross reactivity to other species (Jackson Immuno-Research), were used for cell visualization. For preserving fluorescence and for nuclei detection, a drop of VECTASHIELD mounting medium with DAPI was added (Vector). The sections were examined under a Zeiss Laser Scanning Confocal Microscope, using a ×60 magnification lens. The percentage of co-expression/localization was calculated by measuring the ratio between two crossed intensities in a defined area using an Olympus Fluoview FV1000 version 2b.

### Statistical analysis

For measuring the amounts of total N-glycans in serum and for determining the IgG glycan structures and their amounts, individual serum samples of each group were used. The data shown correspond to pooled or single representative experiments, as indicated, and are expressed as mean values ± SEM. Significant differences in results were determined using three t-tests; the Mann-Whitney test, the Welch t-test, and ANOVA, with p<0.05 in the triplet tests being considered as significant. The p value observed for N-glycans was calculated before multiple testing. The p values after multiple testing differed insignificantly compared to the values before. All statistical analyses were performed at the Bioinformatics Core Facility at Ben-Gurion University using Partek® Genomics Suite™ software.

The *in vitro* experiments were repeated at least 3 times with similar patterns of responses. The data shown correspond to pooled or single representative experiments, as indicated, and are expressed as mean values ± SD. Significant differences in results were determined using the two-tailed ANOVA t-test, with p<0.05 being considered as significant.

**Figure 4:**
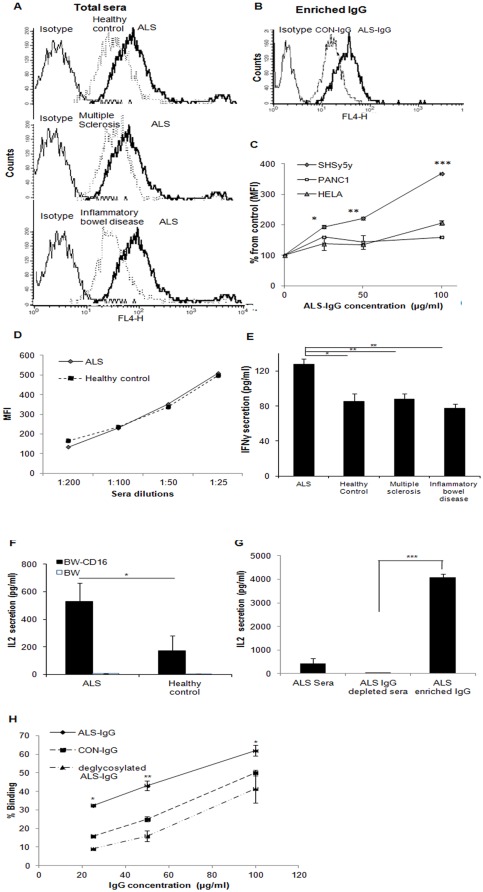
Coupling of IgG to target neuroblastoma and NSC34 cells and to CD16 on effector cells. FACS histograms presenting a shift in binding of serum pools of ALS patients to human neuroblastoma cells in comparison to binding of healthy control (CON), inflammatory bowel disease and multiple sclerosis serum pools to neuroblastoma cells (**A**); FACS histogram presenting shift in binding of purified IgG from serum pools of ALS patients to neuroblastoma cells relative to binding of purified IgG from healthy control (CON) (**B**); Dose-dependent coupling of purified ALS-IgG to human PANC1, HeLa, and neuroblastoma cells performed as described in A(**C**); Mean fluorescent intensity (MFI) calculated relative to control sample containing cells and serum that was free of IgG. Dose-dependent coupling of ALS-IgG to mouse NSC34 cells was performed as described above (**D**); Secretion of IFNγ by enriched human peripheral NK cells in response to interactions with pools of ALS, inflammatory bowel disease patients, patients of multiple sclerosis, and healthy control (CON) sera (**E**); Secretion of IL-2 by BW-CD16 transfectants or BW cells in response to interactions with pools of ALS and healthy control sera (**F**), and in response to interactions with ALS-IgG and ALS IgG-depleted sera (**G**). Comparing the specificity of dose-dependent coupling of PNGase F-treated or untreated IgG of ALS patients and of the IgG of healthy volunteers, to CD16 (**H**). Data represent the mean ± SD of triplicate measurements from independent duplicate experiments. Pools of healthy and patient samples contained a mixture of four individual serum samples with similar glycan amounts represented in peaks 12 and 13. Statistical significance, *** p<0.005, ** p<0.01 and * p<0.05, versus the appropriate controls in each panel.

**Figure 5:**
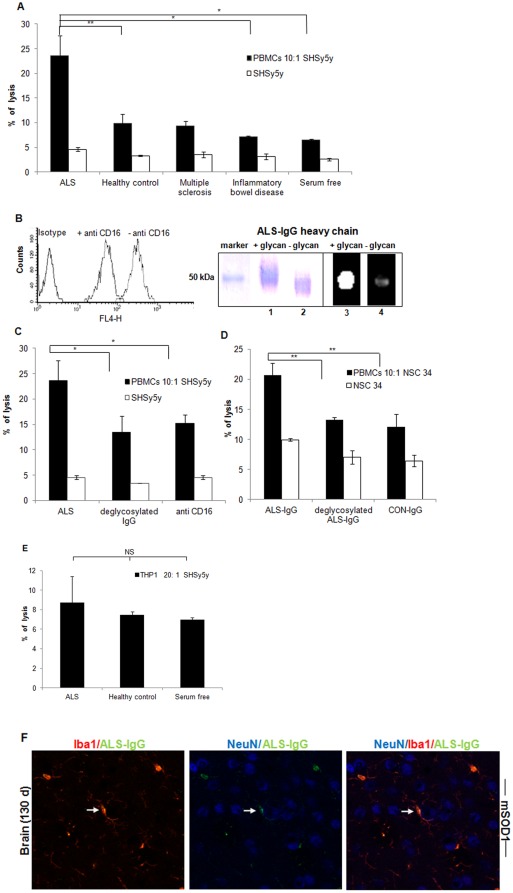
Killing human neuroblastoma or mouse NSC34 cells through the ADCC pathway. ADCC was performed using human neuroblastoma as target cells, PBMCs as effector cells, and pools of serum samples of ALS, healthy control, inflammatory bowel disease patients, and multiple sclerosis patients as IgG sources. The controls contain: neuroblastoma cells incubated with IgG pools from the different serum sources and co-cultures of neuroblastoma cells and PBMCs (**A**). FACS results from PBMCs pre-treated with anti-CD16 antibodies (**B**, left) and the heavy chain of ALS-IgG before and after PNGase-F treatment in SDS-PAGE and Western blot using ECL lectin (**B**, right); ADCC mediated by CD16-blocked effector cells or by ALS-IgG after PNGase-F treatment, as compared to the ADCC against neuroblastoma mediated by unblocked effector cells and untreated ALS-IgG (**C**); Killing of NSC34 cells by ADCC as described above was mediated by intact (untreated) ALS-IgG, PNGase-F treated ALS-IgG and by IgG from healthy controls (**D**). Neuroblastoma lysis by CD32- and CD64-positive THP1 cells was mediated by ALS-IgG, IgG of healthy controls, and in serum free of IgG (**E**). Spontaneous lysis was measured in neuroblastoma or NSC34 cultures. Triple staining of NeuN, Iba1, and ALS-IgG by anti-human IgGs conjugated to FITC demonstrates the localization of intact ALS-IgG in immune synapse (arrow) amongst microglia and neurons (**F**). Data represent the mean ± SD of triplicate measurements from triplicate independent experiments. Pools of healthy and patient samples contained a mixture of four individual serum samples with similar glycan amounts represented in peaks 12 and 13. Statistical significance, ** p<0.01, * p<0.05 and NS (Not significant) is represented versus the appropriate controls in each panel.

## Results

### Sequencing N-glycans derived from intact serum glycoproteins

Based on the presence of various blood substances in the cerebrospinal fluid [22], we hypothesized that the serum substance repertoire of ALS patients might contain uncommon glycoforms. Accordingly, we sequenced total N-glycans derived from serum samples of ALS patients and healthy volunteers. Whole serum N-glycans from 19 patients were fractionated by quantitative NP-HPLC, according to glucose units (GU). The thirteen fractions observed were numbered (**Fig. S1**) and each was pooled and subsequently digested by exoglycosidases, with structural assignments being made using database-matching, combined with MALDI-TOF MS. The results from the pooled fractions were compared to those of control samples pooled from fractionated N-glycans of 24 healthy volunteer sera. The results revealed a similar number of fractions and glycan structures in both patient and healthy serum samples. Most of the fractions contained 3–5 glycan structures, namely bi- or tri-antennary high mannose and complex type structures (**Table S1**). The complex structures were composed of neutral and multi-sialylated glycans. Some of these were core fucosylated, while others bore bisecting N-acetyl glucosamine (GlcNAc) or sialyl-Le^x^ epitopes. Despite the fact that many structures were found to be capped by sialic acid residues, separation by weak ion-exchange HPLC according to charge failed to provide full sequencing information due to partial glycan separation (data not shown). Thus, the glycans partially sequenced by NP-HPLC separation were next subjected to sample fractionation.

In addition to the full sequencing of serum glycans, N-glycan amounts from individual samples were calculated by dividing areas under specific peaks to total peak area measured in each HPLC spectrum. Following t-test analyses of the individual samples, 4 fractions met statistical criteria (P<0.05) and were selected as candidate disease glycans (**Table 1** **, Table S2**). Two of the fractions, peaks 10 and 13, contained abundant bi- and tri-antennary glycans that included up to two sialic acid residues or sialyl-Le^x^ epitopes. These structures were significantly up-regulated in ALS samples. The two other fractions, peaks 6 and 8, were rich in core fucosylation and galactosylation and were plentiful in healthy samples. The unique glycans detected upon individual sample analysis were correlated with glycan alterations found in the pooled samples. Thus, this pilot study indicates that glycans are potential candidate markers of ALS. However, full serum glycome analysis may not be successful in revealing differences in the glycans of specific glycoproteins which might play a central role in disease progression.

### Sequencing the N^297^ glycans of enriched IgG

The involvement of the IgG Fc domain in IgG uptake was suggested in an ALS animal model [10]. Accordingly, IgG molecules were purified from individual serum samples of 19 ALS, 22 inflammatory bowel disease, and 6 multiple sclerosis patients, and from 24 healthy controls. N-glycans were released from a total of 71 individual samples and separately analyzed by NP-HPLC and MALDI-TOF MS. Results showed similar bi-antennary complex structures (**Fig. S2, Table S2, Table S3**). T-test analyses of glycan amounts in ALS and healthy subjects revealed two structures in amounts that met our statistical criteria as being unique N^297^ glycans (**Table 2**). Similar Fc glycans purified from healthy control IgG were observed, as compared to ALS-IgG, except for one significant difference, namely, a galactosylated structure with a bisecting GlcNAc lacking the core fucose (A2BG2, peak no 7) was doubled in ALS-IgG. As well, the A2BG2 structure was significantly (P<0.015) up-regulated in ALS-purified IgG, as opposed to in purified IgG from inflammatory bowel disease or multiple sclerosis patients (**Fig. 1A**). Moreover, **Fig. 1A** illustrates the amounts of A2BG2 structure in pools of healthy controls, ALS, inflammatory bowel disease, and multiple sclerosis patients. As can be seen, when pooling the individual samples from each examined group and analyzing by NP-HPLC, similar amounts of A2BG2 were observed as compared to individual samples. To diminish differences of glycan structural alterations with respect to other clinical parameters such as, sex and age, we regrouped the serum sample by gender or age and performed ANOVA t-test analysis. **Fig. 1B and 1C** show that A2BG2 amount does not change with respect to gender or age, respectively. Hence for the next experiments, designed to elucidate A2BG2 role in determining IgG activity, we used pooled samples. However, as indicated in **Table 2**, double and triplicate glycan amounts represented in peaks 12 and 13 respectively, were further observed in patient samples, but found to be insignificant as compared to healthy samples. Accordingly, patient and healthy samples with similar glycan amounts represented in peaks 12 and 13 were selected to assemble the pools.

### Abundant expression of CD16 and its co-expression with microglia of G93A-SOD1 brain tissue

The indicated Fc glycan A2BG2 is known to increase IgG coupling with CD16 on effector cells, thereby enhancing ADCC. Therefore, in order to illustrate the expression of CD16, brain and spinal cord tissues of 130-day and 75-day old G93A-SOD1 mice and wild-type littermates were sectioned. Staining with anti-CD16 antibodies showed that CD16 was abundantly expressed by brain and spinal cord tissues of 130- and 75-day old mSOD1 mice in comparison to wild-type mice (**Fig. 2A–B**). However, CD16 expression in the brain tissue was enhanced in comparison to the spinal cord tissue. Presumably, the level of CD16 depends on the number and type of cells expressing this receptor. Therefore, we next examined CD16 co-localization to microglia by staining with CD16 and microglial markers. Co-expression of CD16 with microglia cells was detected in the brain tissue sections (**Fig. 2A**). Microglia in wild-type brain tissue expressed low levels of CD16 (yellow spots) and changed their morphology from ramified to amoeboid in mSOD1 mice, whereas CD16 was expressed at the branches and around the cellular body (the small images of wild-type and its counterpart mSOD1 brain tissues, respectively). In contrast, in spinal cord tissues of 130- and 75-day old mSOD1 mice, the CD16 receptor was poorly co-expressed with microglia. The quantitative analysis summarizes the data shown in figure 2A–B and shows that CD16 was significantly over-expressed in mSOD1 brain and spinal cord tissues in comparison to the wild-type counterparts (**Fig. 2C**). Specifically, CD16 was abundantly co-expressed with microglia in brain tissue of mSOD1 mice in comparison to that observed in wild-type brain tissue or in spinal cord tissues of mSOD1 mice (**Fig. 2D**).

### ALS-IgG co-localized with CD16 and microglia

To investigate whether the elevated amounts of A2BG2 glycan enhance co-localization of ALS-IgG with CD16 and with microglia, pools of IgG purified from healthy candidates or from ALS patients, with and without the Fc N-glycans were used on brain and spinal cord tissue sections of mSOD1 mice and wild-type littermates. Intact ALS-IgG, with the Fc glycans, were significantly found in 130-day old mSOD1 brain and spinal cord tissues as opposed to lower localization of PNGase-F-treated ALS-IgG and of IgG of healthy candidates in mSOD1-matched tissues (**Fig. 3A, B**). Furthermore, localization of ALS-IgG in the wild-type brain and spinal cord tissues was lower than observed in mSOD1 tissues (**Figure 3A, B**). Similar results were observed in spinal cord tissue sections of 75-day old mSOD1 mice (**Fig. S3A**). However, at the early stage of the disease (75 days), less ALS-IgGs were detected on the sections in comparison to the final stage of the disease (130 days).

Co-localization of ALS-IgG with CD16 in mSOD1 brain tissue was largely detected on cell surface and branches, apparently of microglia cells (**Fig. 3A, C**). Double staining verified that intact ALS-IgG co-localized with mSOD1 microglia cells, where the IgG positioned around the microglia cellular body (**Fig. 3D**) and branches. Approximately, 65% of the total number of microglia per field bound ALS-IgG. However, ALS-IgG were also detected near the nuclei of other cells, especially in spinal cord tissues. These results are in contrast to those observed when using PNGase-F-treated ALS-IgG or IgG of healthy samples, on mSOD1-matched sections (**Fig. 3A, C**).

Staining of neurons and ALS-IgG indicated that ALS-IgG were co-localized with neurons in brain and spinal cord tissues of 130-day old mSOD1 mice (**Fig. 3E**) and in spinal cord tissues of 75-day old mSOD1 mice (**Fig. S3B**).

Taken together, our results related to the increase in amount of IgG-A2BG2 glycoform in ALS sera, the CD16 over-expression in tissue sections of G93A-SOD1 mouse brains and spinal cords, and the co-localization of ALS-IgG with CD16 and brain microglia, suggest that N-glycans of ALS-IgG may be involved in ADCC reactions. Therefore, our next step was to illustrate the ALS-IgG involvement in ADCC.

### Binding of serum IgG to a human neuroblastoma and mouse NSC34 cell lines and to lymphocyte CD16

Initially, we verified that neurons can serve as antigenic targets for ALS-derived IgG [23]–[25], and that they poorly bind IgG from healthy controls or from inflammatory bowel disease or multiple sclerosis patient samples. As such, neuroblastoma cells blocked by anti-CD16/CD32 antibodies were incubated with pools of serum samples or with purified IgG and then assessed by FACS. The blocking step was performed only in the experiments of binding IgG to the target cells in order to reduce unspecific binding. The binding of either purified or unpurified ALS-IgG to the surface of the neuroblastoma cells was elevated, in comparison to such binding by pools of IgG from healthy control (**Fig. 4A, B**), or by IgG pools from inflammatory bowel disease (**Fig. 4A**) or from multiple sclerosis patients (**Fig. 4A**). Comparing the specificity of ALS-IgG binding by neuroblastoma, HeLa and PANC1 cells revealed significant differences between neuronal and non-neuronal cells (**Fig. 4C**). To confirm the latter result but with the appropriate target cell, we repeated the binding experiments using neuroblastoma-spinal cord motoneuron hybrid cell line, NSC34 exposed to different serum concentration. As opposed to neuroblastoma, NSC34 cells bound ALS-IgG in a similar manner as they bound IgGs from healthy controls (**Fig. 4D**). This is particularly important when demonstrating the cytotoxic effects of IgGs via their coupling to CD16 in the ADCC reaction.

Hence, we next investigated whether the elevated amounts of A2BG2 glycan in the Fc domain found in ALS-IgG increase the affinity of these antibodies to CD16 [2], [3] by measuring cytokine production and the percentage of IgG binding to CD16. Pools of serum samples from healthy controls, ALS, from patients with inflammatory bowel disease and from multiple sclerosis patients, containing similar concentrations of IgG were incubated with purified human peripheral NK cells for 18 h. In addition, pools of serum samples from healthy controls and ALS or ALS-IgG and ALS IgG-depleted sera were incubated with BW-CD16-transfected or normal BW cells [26], for 18 h. NK cells containing CD16 and BW-CD16 transfectants produced IFNγ and IL-2, respectively, in response to Fc ligand coupling. ELISA results demonstrated that NK cells were activated by ALS patient sera to produce augmented amounts of IFNγ, while inflammatory bowel disease patient, multiple sclerosis patient or healthy control sera induced lower IFNγ production (**Fig. 4E**). Moreover, more than double the amount of IL-2 was produced by BW-CD16 transfectants in response to ALS patient sera, as compared to healthy control sera, while normal BW cells incubated with any sera did not produce IL-2 (**Fig. 4F**). In response to purified IgG, BW-CD16 transfectants produced more than 8-fold amount of IL-2, whereas negligible amounts of IL-2 were produced in response to ALS IgG-depleted sera (**Fig. 4G**). By FACS and several dilutions, examination of the specific coupling to CD16 of PNGase F-treated or untreated ALS-IgG or of IgG of healthy volunteers, revealed significant differences between the IgG containing the A2BG2 glycan to those lacking this glycan (**Fig. 4H**).

### Loss of human neuroblastoma and mouse NSC34 cells through the ADCC pathway

To illustrate the involvement of ALS-derived IgG in mediating ADCC, cytotoxic assays were performed using human neuroblastoma or mouse NSC34 as target cells, PBMCs as effector cells and pools from healthy controls, ALS, inflammatory bowel disease, and multiple sclerosis patients as IgG sources. A lysis rate of 25% was mediated by IgG from ALS patient sera (**Fig. 5A**), while healthy control, inflammatory bowel disease patient and multiple sclerosis patient sera mediated cytotoxicity of less than 10% (**Fig. 5A**). A lysis rate of 7% was measured in samples of neuroblastoma cells co-cultured with PBMCs and values of less than 5% when neuroblastoma cells were incubated with IgG samples to control for spontaneous cell lysis.

To illustrate the involvement of CD16 in the ADCC reaction mediated by ALS-IgG, PBMCs were blocked with anti-CD16 antibodies and IgG were purified from pool samples. FACS results show a significant decrease in coupling of ALS-IgG to PBMCs, as detected by secondary antibodies (**Fig. 5B left panel**). Using the CD16-blocked PBMCs in an ADCC reaction against neuroblastoma cells led to a 40% reduction in the cytotoxic response against the target cells, in comparison to cell lysis with unblocked PBMCs (**Fig. 5C**). To illustrate the effect of the A2BG2 glycoform on neuroblastoma lysis, N-glycans were removed from ALS-IgG by PNGase F treatment and the heavy chain was assessed by SDS-PAGE and Western blot using ECL lectin conjugated to FITC. N-glycans with galactose residues have high affinity to the ECL lectin [27]. As can be seen, the heavy chain of ALS-IgG before PNGase F treatment migrated with an average apparent molecular weight of 50 kDa (lane 1) and was well labeled in the Western blot protocol (lane 3) (**Fig. 5B** **right**). After PNGase F treatment, however, the heavy chain band was shifted to an apparent molecular weight lower than 50 kDa (lane 2), with the fluorescent intensity associated with the immunoblot being quenched (lane 4) (**Fig. 5B** **right**). When using ALS-IgG after PNGase F treatment in an ADCC response against neuroblastoma cells, a two-fold decrease in lysis was noted compared to using ALS-IgG bearing N-glycans (**Fig. 5C**). In comparable experiments, killing of NSC34 target cells by PBMCs was conducted using intact ALS-IgG, ALS-IgG treated with PNGase F and IgG from healthy control samples. A similar killing pattern of NSC34 cells as measured for neuroblastoma cells was mediated by intact ALS-IgG, the treated IgG and those from healthy controls (**Fig. 5D**). Furthermore, opsonizing of ALS-IgG by CD64 and CD32, which either leads to target cell lysis was reversed by using THP1 cells expressing CD32 and CD64 but not CD16 [28], as an alternative to NK cells in the cytotoxic assay (**Fig. 5E**). These observations indicate that CD16 on NK cells and the A2BG2 in ALS-IgG are involved in neuroblastoma and NSC 34 cell loss.

Finally, to demonstrate the feasibility of *in vivo* ADCC, we studied the localization of intact IgG derived from ALS patients with both neuron and microglia of mSOD1 brain tissue, by triple staining. Several ALS-IgG molecules were found to be located in the immune synapse between microglia and the neuron, suggesting the occurrence of ADCC (**Fig. 5F**). In contrast, such localization was rarely observed in matched-sections when PNGase-F-treated ALS-IgG were used (data not shown). Accordingly, the Fc glycans are involved in IgG deposition in the brain of an ALS animal model and plausibly take part in *in vivo* ADCC.

## Discussion

Previous studies have shown that the glycan chain synthesis machinery is highly sensitive to the biochemical environment and can change during the course of a disease [29]. In neurodegenerative disorders such as Alzheimer's and Creutzfeldt-Jakob diseases, it was found that the glycosylation pattern of several glycoproteins, such as reelin, or acetylcholinesterase, is associated with disease pathogenesis [30], [31]. Thus, the altered expression of glycoproteins in the sera or cerebrospinal fluid of patients with ALS [32], [33] encouraged us to utilize a glycomics approach in the hope of finding ALS-unique glycans in patient sera.

The present pilot study analyzes the N-glycome of both individual and pooled sera, reflecting changes in N-glycan quantities rather than in unique structures. We observed that sialylated glycans are significantly increased in ALS patient sera while fucosylated glycans are significantly decreased, as compared to healthy control sera. Our results are in accordance with previous studies showing increased glycan sialylation in breast and ovarian cancer patient sera [34], [35]. The results are, however, opposed to studies that showed over-expression of fucosylated glycans in sera of hepatocellular carcinoma and pancreatic cancer patients [36], [37]. Although similar glycan aberrations can be observed in totally different types of diseases, the alterations detected in ALS patient sera represent the first step in finding candidate disease biomarkers. Identifying such markers depends on a correlation with disease severity as measured by alternative assays, which would be necessary to improve specificity and sensitivity. Such identification also depends on a large scale study. Whereas serum N-glycans can be considered as source biomarkers for disease diagnosis and prognosis, profiling the glycans of specific glycoproteins under disease conditions might also reveal the involvement of glycans or glycoproteins in disease pathogenesis.

Based on the results of the N-glycome analysis reported, we analyzed IgG-Fc glycans from patient or healthy sera, using a second and more specific strategy [38]. As IgG are the most abundant glycoproteins in serum, they were predicted to contain altered fucosylated and/or sialylated glycans, as observed in the serum N-glycome of ALS patients versus healthy volunteers. Also, changes in fucosylation and sialylation in the Fc domain of IgG were previously associated with ADCC and the inflammation process, respectively [39]–[41]. Moreover, IgG molecules which contain variable domains against neurons [24], [25] were detected in spinal cord motor neurons and in pyramidal cells within the motor cortex of ALS patients. It was also found that the IgG-Fc domain is necessary to the IgG uptake by motor axon terminals [7]. Therefore, it was suggested that IgG-Fc plays a role in the pathogenesis of ALS and that ALS might be an autoimmune disease [23], [42]. Indeed, changes in IgG-Fc glycans have been associated with autoimmunity [3]. Thus, two autoimmune diseases were utilized here as comparable IgG sources, one a neurodegenerative disease, multiple sclerosis, and the other an inflammatory bowel disease of the gastric system. We demonstrated the expression of A2BG2 in the Fc domain of ALS-derived IgG. This glycan was not detected in healthy control-derived IgG and detected to low extent in IgG derived from patients with inflammatory bowel disease or multiple sclerosis. In these diseases as compared to healthy conditions, the inflammation determines the expression of glycosyltransferases in B cells, thus the changes in the Fc glycans. In contrast, a similar structure containing core fucose was largely found in healthy control-derived IgG. This is consistent with previous works showing high core fucosylation in normal human IgG [43], [44]. Therefore, A2BG2, which reflects, in part, the reduced fucosylation observed in the serum N-glycome of ALS patients, is an appropriate candidate for further exploration of potential biomarkers for ALS diagnosis. Notably, a comparison between the different IgG glycan sources indicated that a significant number of glyco-variants associated with autoimmunity [45] were not found in ALS-IgG (data not shown).

Previously, it was shown that enhanced ADCC against cancer cells could be achieved by engineered bisecting GlcNAc IgG glycoforms [46], [47], while weak ADCC was observed by IgG glycoforms rich in core fucosylation [41]. The Fc glycans determine the affinity to the activating receptor, FcγRIII (CD16). For example, core fucosylation of the N^297^-Fc glycans decreases the affinity to CD16 by 50-fold [41]. Therefore, we hypothesized that considerable amounts of A2BG2 in ALS-IgG would advance a higher ADCC. We pooled samples of ALS-IgG or control IgG with similar glycan amounts, excluding the A2BG2, and indeed showed that ALS-derived IgG better mediates ADCC against neuroblastoma and hybrid of neuroblastoma-spinal cord motoneuron cells by human peripheral NK cells, and that the lysis was not a consequence of phagocytosis. Also, IgG coupling from the healthy, the inflammatory bowel disease and the multiple sclerosis patients led to a lower degree of neuroblastoma lysis, indicating that cytotoxicity is specifically associated with the ALS patient IgG. Pre-treatment of PBMCs with anti-CD16 antibodies remarkably reduced the ADCC reaction mediated by ALS-IgG, while an 18 h incubation of ALS-IgG with CD16-transfected BW cells or peripheral NK cells induced IL-2 or IFNγ, respectively. Furthermore, removing N-glycans from the Fc of ALS-IgG by specific cleavage also reduced the ADCC reaction. Our results are in accordance with previous *in vitro* studies, providing evidence for ALS-IgG but not for control IgG mediating of neuron loss [25], [48]. These studies, however, showed the effects of IgG on neuron apoptotic death by incubating the cells with only IgG in long-term cultures. We suggest a pathway for neuron loss through ADCC and show by *in vitro* study a plausible contribution of the IgG A2BG2 glycan to this pathway.

Staining brain tissue sections derived from 130-day old G93A-SOD1 mice by ALS-IgG or healthy control IgG, and staining for CD16 on microglia and neurons supported our hypothesis for neuron loss through ADCC that can be mediated by IgG with the A2BG2 glycan. CD16 was more abundantly expressed in mSOD1 brain than in non-SOD1 littermates and co-expressed with microglia, revealing the capability of this receptor to bind IgG with A2BG2 glycan at a high frequency. Indeed, ALS-IgG molecules with the Fc-glycan had a higher incidence of binding to CD16 than those without the glycans or those from healthy samples, and they localized amongst microglia and neurons. These findings are in line with previous reports showing the Fc-domain contribution to ALS-IgG uptake by motor axon terminals and the Fcγ receptor role in reducing ALS-IgG uptake, and attenuating accumulation of intracellular calcium and acetylcholine release by neurons [7]. Staining of spinal cord tissues supported most of the findings revealed in brain tissue; excluding the co-localization of CD16 with microglia cells. It is plausible that in the spinal cord other CD16 bearing populations of infiltrate immune cells [14]–[15] take part in the ADCC. Nevertheless, CD16 affects the IgG position in the brain and spinal cord more than does the neuron antigenic domain. This was further documented in the spinal cord of 75-day old G93A-SOD1 mice, which are at the earlier stage of the disease. The lower levels of ALS-IgG that were detected on spinal cord tissue sections of 75-day in comparison to 130-day old mSOD1 mice, can point to the role of IgG glycans in the pathogenesis of ALS, as at the earlier stage of the disease the pathological features are not solely a consequence of neurodegeneration as in the final stage of the disease.

The antigenic domains found in auto-antibodies of ALS patients include Fas, neurofilaments, voltage-gated Ca^2+^ channels, GM1 gangliosides [48]–[50] and the gangliosides, GD-2 and GD-3 [51]. It can be assumed that the frequent presence of an IgG fraction containing a neuron-antigenic domain and an Fc domain carrying the A2BG2 glycan in ALS blood induces neuron death by ADCC. Accordingly, our results suggest an involvement of the activating receptor FcγRIII in the neuron loss, caused by an increase in the affinity of ALS-IgG to FcγRIIIA via the Fc glycan with its bisecting GlcNAc lack of core fucose.

To summarize, in addition to the more sialylated and less fucosylated glycans revealed in ALS patient sera, we have demonstrated herein the involvement of a specific glycan structure in neuroblastoma and NSC34 cell lysis. IgG previously found to alter neuron functions, thereby determining neuron survival, were shown here to potentially play a role in the cytolytic response against neurons mediated by the Fc domain. We therefore, propose an involvement of IgG Fc-glycan in neuron death in ALS, which is governed by CD16 on microglia or infiltrating immune cells. As well, we propose that glycans of IgG from ALS patients may serve as a biomarker for the disease.

## Supporting Information

Figure S1 **Fractions of whole serum N-glycans.** The total N-glycans from individual samples of ALS patients or healthy volunteers were fractionated by quantitative normal phase HPLC, according to glucose units (GU). The thirteen observed fractions were numbered, and each was pooled and subsequently digested by exoglycosidase to determine glycan structures and amounts.
(DOC)

Figure S2 **Sequential exoglycosidase digestions of glycans released from normal human serum IgG and measured by NP-HPLC.** The IgG glycan pool from individual samples (undigested sample) was incubated sequentially with Arthrobacter ureafaciens sialidase (ABS), bovine testes β-galactosidase (BTG), Jack bean β-hexosaminidase (JBH) and Charonia lampas α-fucosidase (BKF). The figure panel shows the HPLC separation of normal IgG glycans and the glycan structure symbols.
(DOC)

Figure S3 **ALS-IgG co-localized with CD16 in spinal cord tissues of 75-day old G93A-SOD1 mice.** Representative confocal microscopic images of spinal cord slices taken from 75-day old G93A-SOD1 mice and age-matched littermates stained for CD16, human IgG, and counterstained with nuclear DAPI. Localization of ALS-IgG before and after PNGase-F treatment and of healthy control-IgG in wild-type and mSOD1 spinal cord tissues (**A**) and co-localization of intact ALS-IgG with NeuN (neurons) in mSOD1 spinal cord tissues (**B**).
(DOC)

Table S1 **Profiles of total N-Glycans derived from pooled sera of ALS patients.** Profiles were observed for both pooled or individual sera of ALS patients and healthy control candidates by using normal phase HPLC and MALDI-TOF MS methods.
(DOC)

Table S2 **Glycan structures.**
(DOC)

Table S3 **Profiles of N^297^-Glycans derived from sera of ALS patients.** Profiles were observed for individual sera of ALS, patients with inflammatory bowel disease, multiple sclerosis patients, and healthy control candidates by using normal phase HPLC and MALDI-TOF MS methods.
(DOC)
