# Supplementary figures and images for: Glycans in Sera of Amyotrophic Lateral Sclerosis Patients and Their Role in Killing Neuronal Cells
Source: PLoS One. 2012 May 30;7(5):e35772. doi: 10.1371/journal.pone.0035772 (PMC3364259; doi:10.1371/journal.pone.0035772)

**
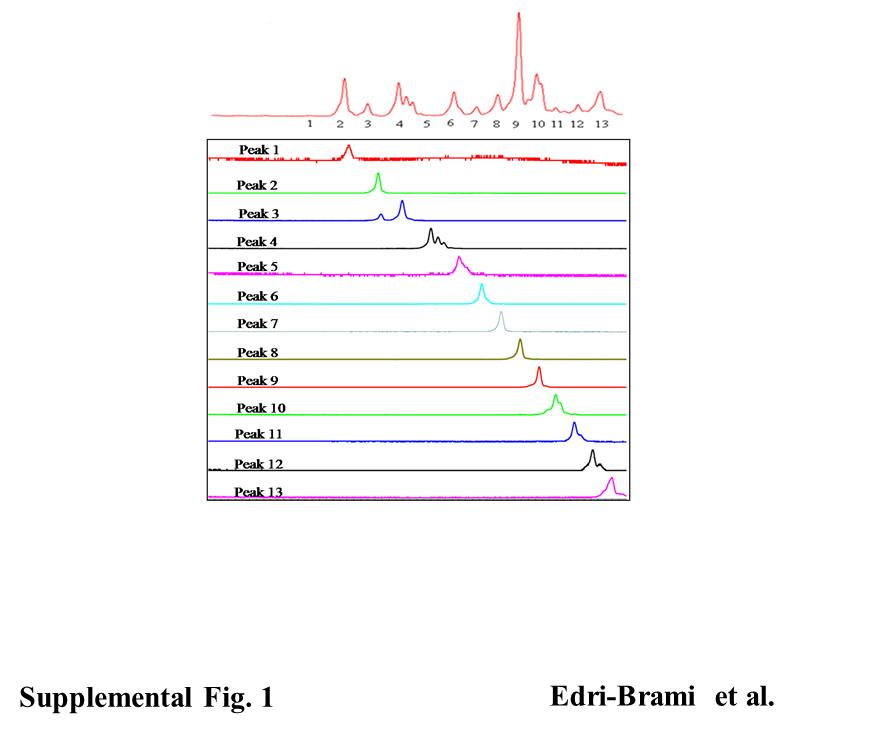
**

Supplement: Figure S1 — Fractions of whole serum N-glycans. The total N-glycans from individual samples of ALS patients or healthy volunteers were fractionated by quantitative normal phase HPLC, according to glucose units (GU). The thirteen observed fractions were numbered, and each was pooled and subsequently digested by exoglycosidase to determine glycan structures and amounts. (DOC) [file pone.0035772.s001.doc]

**
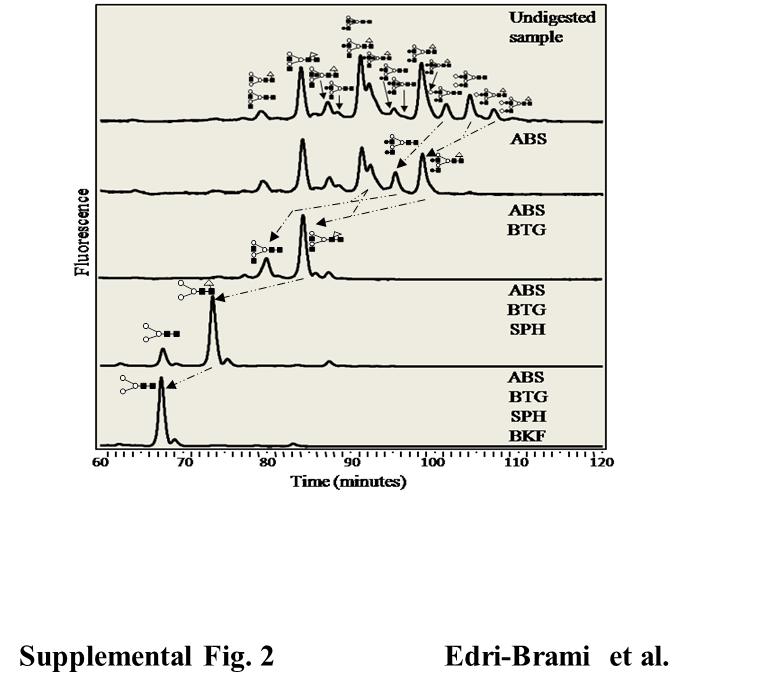
**

Supplement: Figure S2 — Sequential exoglycosidase digestions of glycans released from normal human serum IgG and measured by NP-HPLC. The IgG glycan pool from individual samples (undigested sample) was incubated sequentially with Arthrobacter ureafaciens sialidase (ABS), bovine testes β-galactosidase (BTG), Jack bean β-hexosaminidase (JBH) and Charonia lampas α-fucosidase (BKF). The figure panel shows the HPLC separation of normal IgG glycans and the glycan structure symbols. (DOC) [file pone.0035772.s002.doc]

**
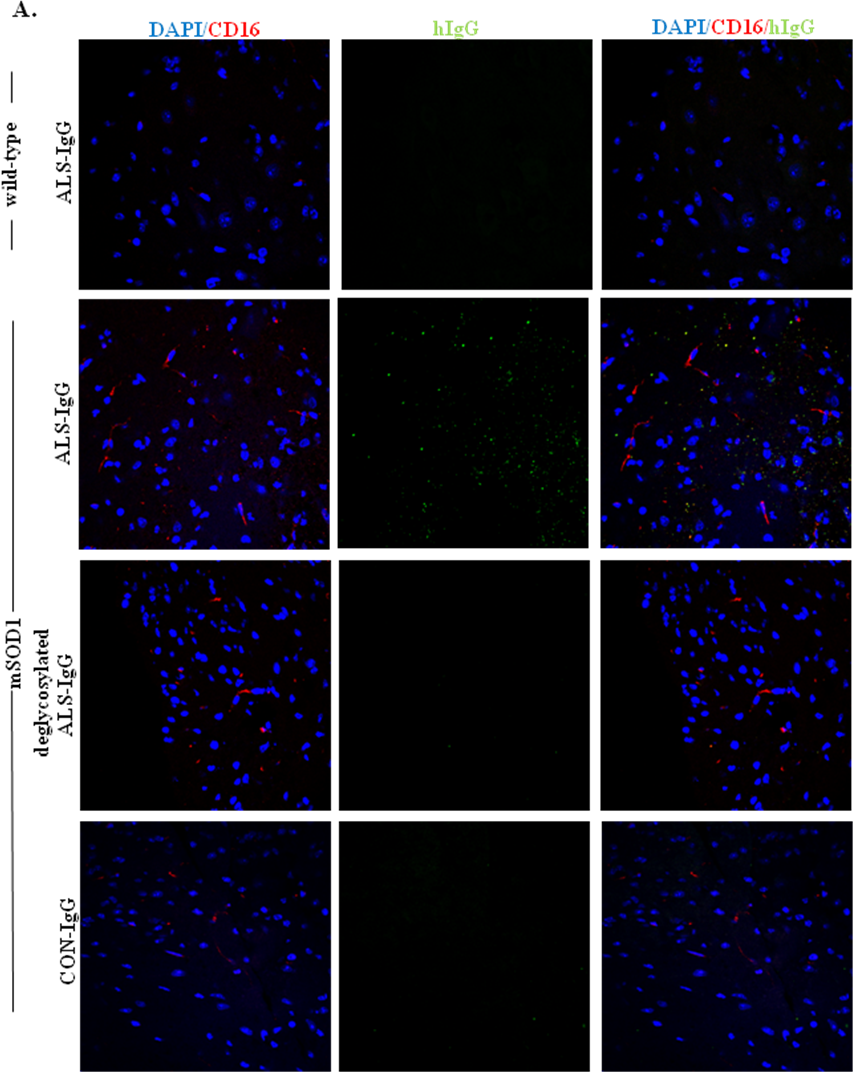
**

**
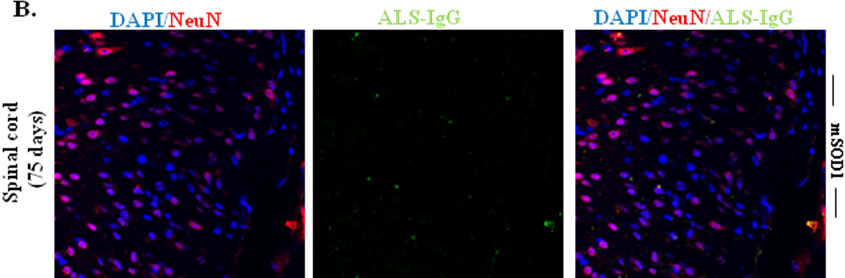
**

Supplement: Figure S3 — ALS-IgG co-localized with CD16 in spinal cord tissues of 75-day old G93A-SOD1 mice. Representative confocal microscopic images of spinal cord slices taken from 75-day old G93A-SOD1 mice and age-matched littermates stained for CD16, human IgG, and counterstained with nuclear DAPI. Localization of ALS-IgG before and after PNGase-F treatment and of healthy control-IgG in wild-type and mSOD1 spinal cord tissues (A) and co-localization of intact ALS-IgG with NeuN (neurons) in mSOD1 spinal cord tissues (B). (DOC) [file pone.0035772.s003.doc]

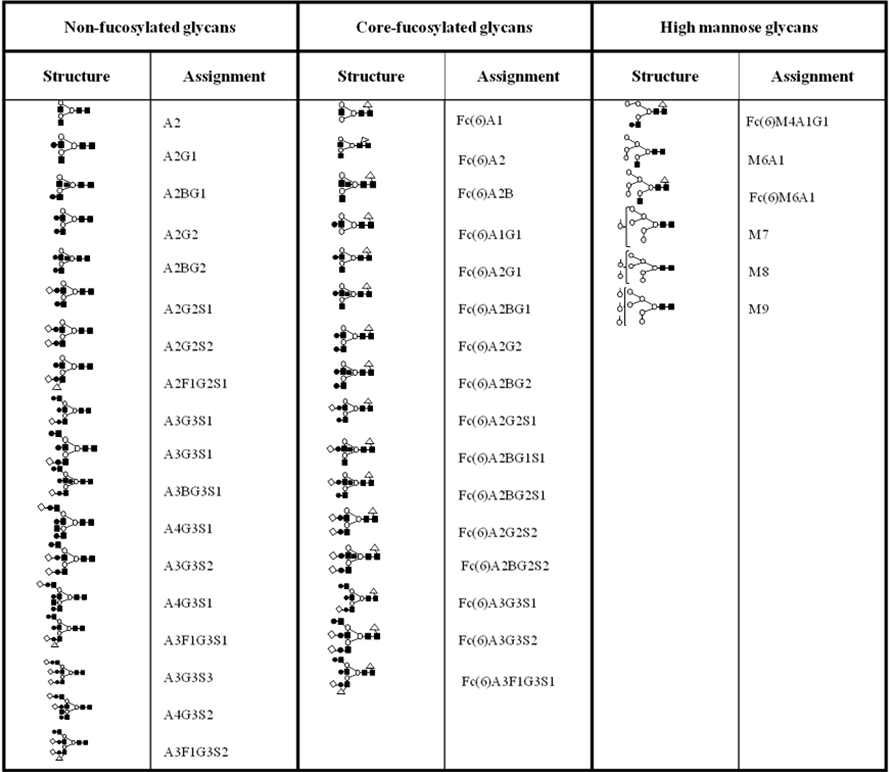

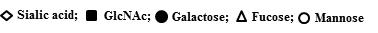
**Table S2.** Glycan structures

Supplement: Table S2 — Glycan structures. (DOC) [file pone.0035772.s005.doc]
