# Supplementary material for: Glycans in Sera of Amyotrophic Lateral Sclerosis Patients and Their Role in Killing Neuronal Cells
Source: PLoS One. 2012 May 30;7(5):e35772. doi: 10.1371/journal.pone.0035772 (PMC3364259; doi:10.1371/journal.pone.0035772)

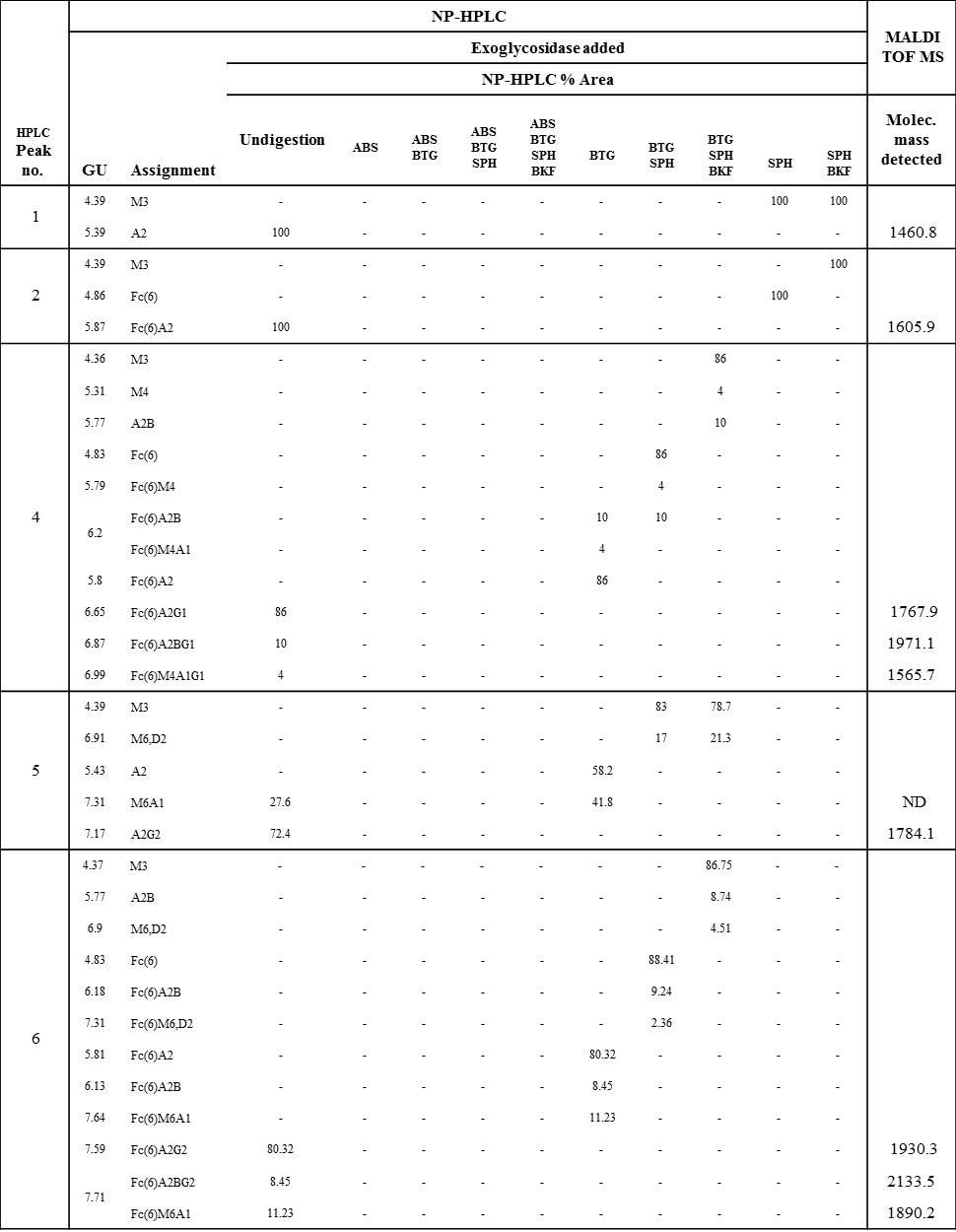
 **Table S1.** Profiles of total N-Glycans derived from pooled sera of ALS patients. Profiles were observed for both pooled or individual sera of ALS patients and healthy control candidates by using normal phase HPLC and MALDI-TOF MS methods.


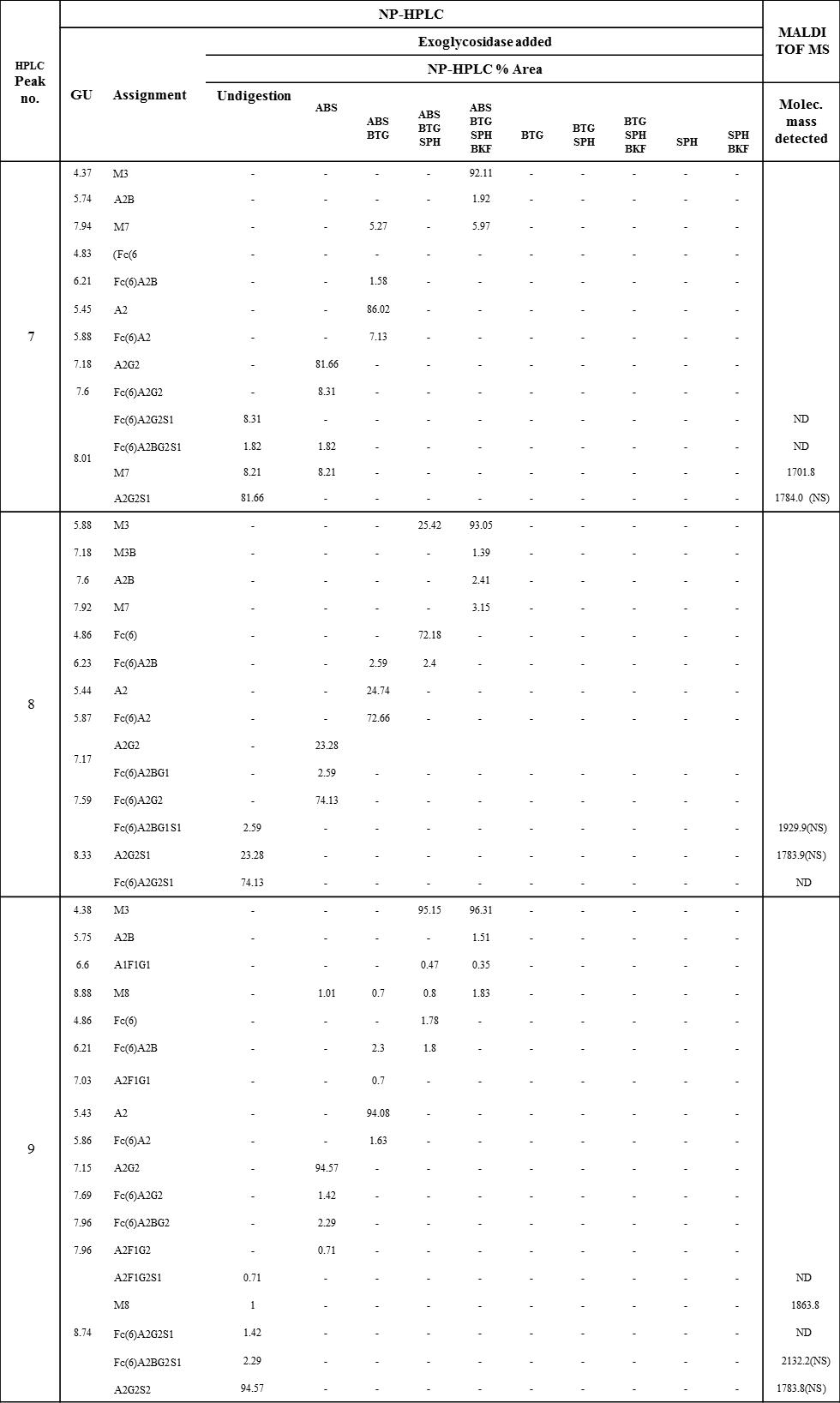


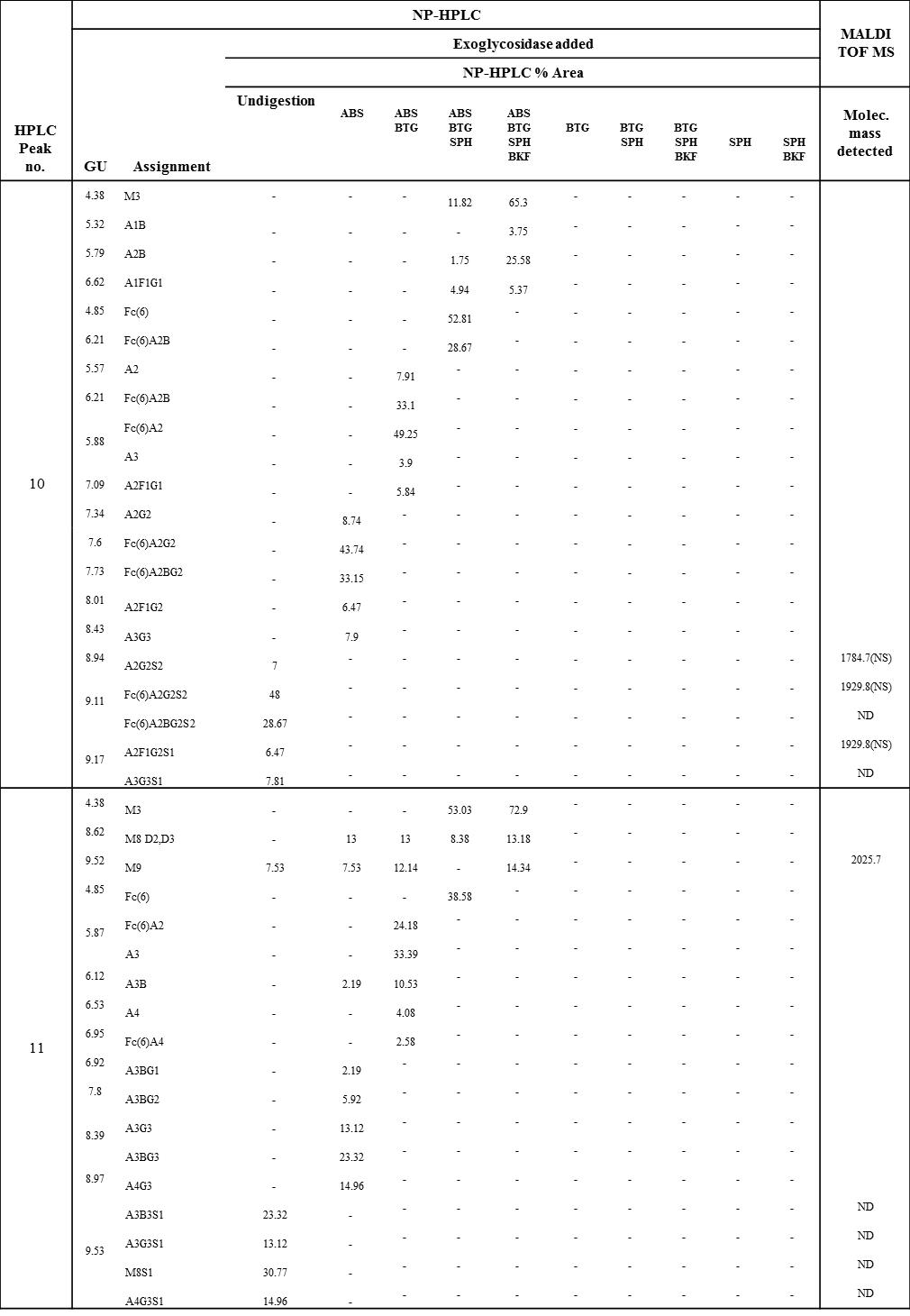


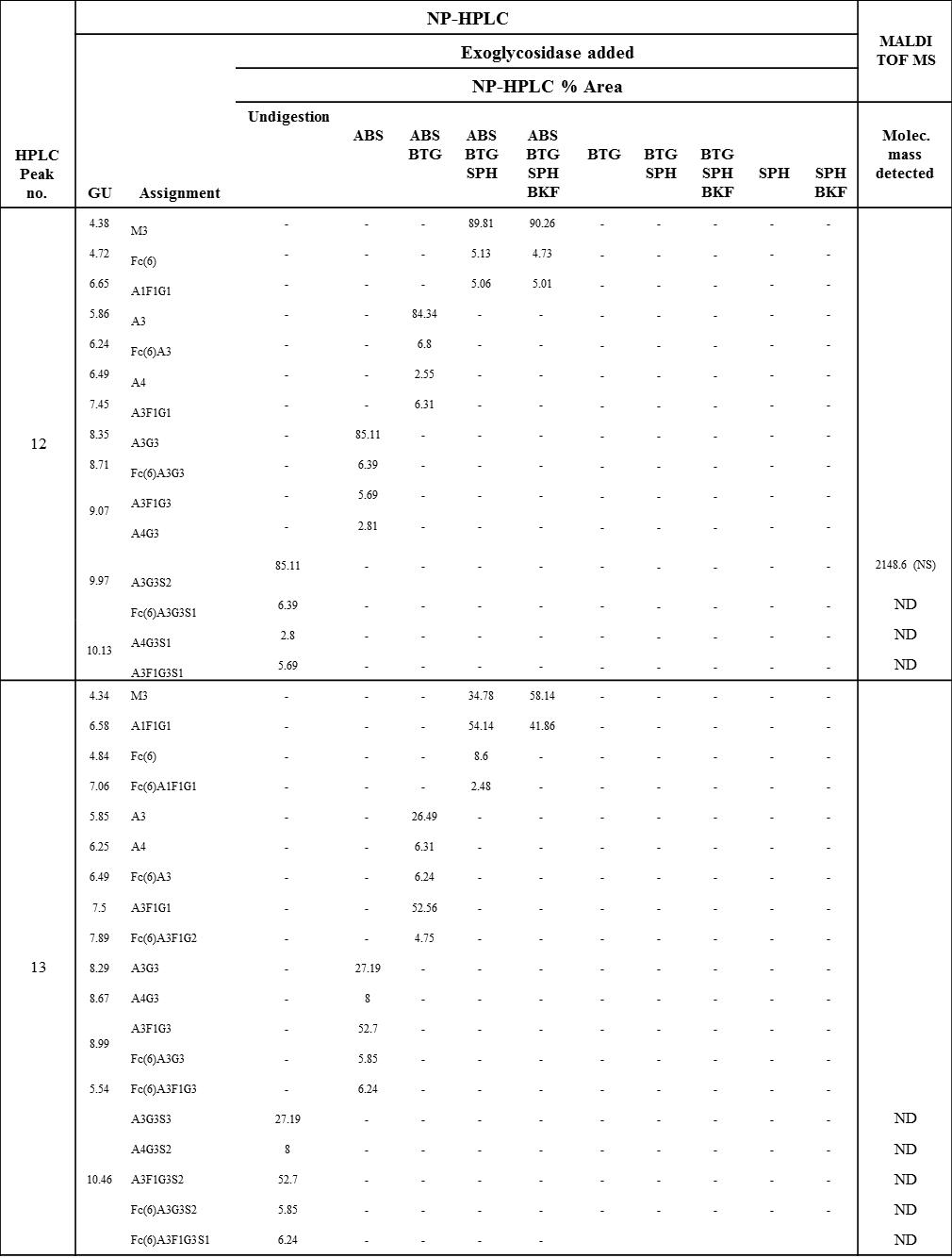

Supplement: Table S1 — Profiles of total N-Glycans derived from pooled sera of ALS patients. Profiles were observed for both pooled or individual sera of ALS patients and healthy control candidates by using normal phase HPLC and MALDI-TOF MS methods. (DOC) [file pone.0035772.s004.doc]
