# Supplementary material for: Glycans in Sera of Amyotrophic Lateral Sclerosis Patients and Their Role in Killing Neuronal Cells
Source: PLoS One. 2012 May 30;7(5):e35772. doi: 10.1371/journal.pone.0035772 (PMC3364259; doi:10.1371/journal.pone.0035772)
Supplement: Table S3 — Profiles of N297-Glycans derived from sera of ALS patients. Profiles were observed for individual sera of ALS, patients with inflammatory bowel disease, multiple sclerosis patients, and healthy control candidates by using normal phase HPLC and MALDI-TOF MS methods. (DOC) [file pone.0035772.s006.doc]

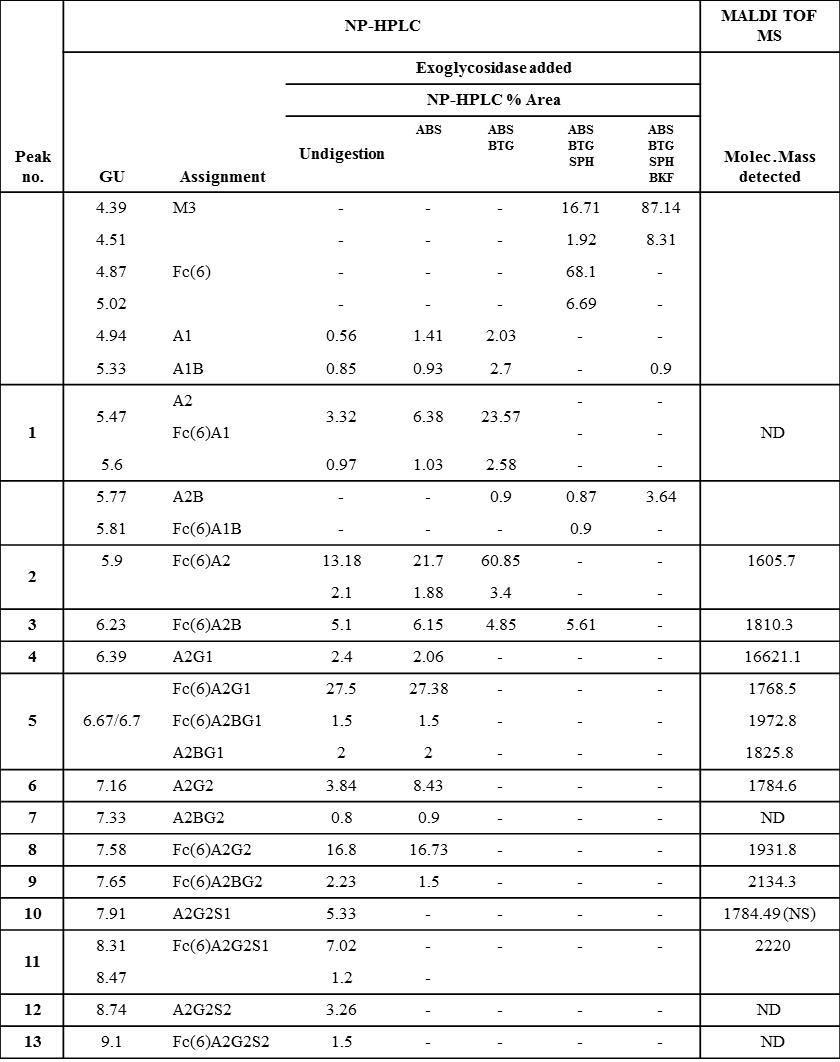
**Table S3.** Profiles of N^297^-Glycans derived from sera of ALS patients. Profiles were observed for individual sera of ALS, patients with inflammatory bowel disease, multiple sclerosis patients, and healthy control candidates by using normal phase HPLC and MALDI-TOF MS methods.
